# Allergic rhinitis

**DOI:** 10.1186/1710-1492-7-S1-S3

**Published:** 2011-11-10

**Authors:** Peter Small, Harold Kim

**Affiliations:** 1 Sir Mortimer B. Davis Jewish General Hospital, Division of Allergy & Clinical Immunology, Montreal Quebec, Canada; 2 University of Western Ontario, London, Ontario, Canada; 3 McMaster University, Hamilton, Ontario, Canada

## Abstract

Allergic rhinitis is a common disorder that is strongly linked to asthma and conjunctivitis. It is usually a long-standing condition that often goes undetected in the primary-care setting. The classic symptoms of the disorder are nasal congestion, nasal itch, rhinorrhea and sneezing. A thorough history, physical examination and allergen skin testing are important for establishing the diagnosis of allergic rhinitis. Second-generation oral antihistamines and intranasal corticosteroids are the mainstay of treatment. Allergen immunotherapy is an effective immune-modulating treatment that should be recommended if pharmacologic therapy for allergic rhinitis is not effective or is not tolerated. This article provides an overview of the pathophysiology, diagnosis, and appropriate management of this disorder.

## Introduction

Rhinitis is broadly defined as inflammation of the nasal mucosa. It is a common disorder that affects up to 40% of the population [1]. Allergic rhinitis is the most common type of chronic rhinitis, affecting 10 to 20% of the population, and evidence suggests that the prevalence of the disorder is increasing. Severe allergic rhinitis has been associated with significant impairments in quality of life, sleep and work performance [2].

In the past, allergic rhinitis was considered to be a disorder localized to the nose and nasal passages, but current evidence indicates that it may represent a component of systemic airway disease involving the entire respiratory tract. There are a number of physiological, functional and immunological relationships between the upper (nose, nasal cavity, paranasal sinuses, pharynx and larynx) and lower (trachea, bronchial tubes, bronchioles and lungs) respiratory tracts. For example, both tracts contain a ciliated epithelium consisting of goblet cells that secrete mucous, which serves to filter the incoming air and protect structures within the airways. Furthermore, the submucosa of both the upper and lower airways includes a collection of blood vessels, mucous glands, supporting cells, nerves and inflammatory cells. Evidence has shown that allergen provocation of the upper airways not only leads to a local inflammatory response, but also to inflammatory processes in the lower airways, and this is supported by the fact that rhinitis and asthma frequently coexist. Therefore, allergic rhinitis and asthma appear to represent a combined airway inflammatory disease, and this needs to be considered to ensure the optimal assessment and management of patients with allergic rhinitis [1,3].

Comprehensive and widely-accepted guidelines for the diagnosis and treatment of allergic rhinitis were published in 2007 [1]. This article provides an overview of the recommendations provided in these guidelines as well as a review of current literature related to the pathophysiology, diagnosis, and appropriate management of allergic rhinitis.

## Pathophysiology

In allergic rhinitis, numerous inflammatory cells, including mast cells, CD4-positive T cells, B cells, macrophages, and eosinophils, infiltrate the nasal lining upon exposure to an inciting allergen (most commonly airborne dust mite fecal particles, cockroach residues, animal dander, moulds, and pollens). The T cells infiltrating the nasal mucosa are predominantly T helper (Th)2 in nature and release cytokines (e.g., interleukin [IL]-3, IL-4, IL-5, and IL-13) that promote immunoglobulin E (IgE) production by plasma cells. IgE production, in turn, triggers the release of mediators, such as histamine and leukotrienes, that are responsible for arteriolar dilation, increased vascular permeability, itching, rhinorrhea (runny nose), mucous secretion, and smooth muscle contraction [1,2]. The mediators and cytokines released during the early phase of an immune response to an inciting allergen, trigger a further cellular inflammatory response over the next 4 to 8 hours (late-phase inflammatory response) which results in recurrent symptoms (usually nasal congestion) [1,4].

## Classification

Rhinitis is classified into one of the following categories according to etiology: IgE-mediated (allergic), autonomic, infectious and idiopathic (unknown). Although the focus of this article is allergic rhinitis, a brief description of the other forms of rhinitis is provided in Table 1.

**Table 1:** Etiological classification of rhinitis [1]

	Description
**IgE-mediated (allergic)**	• IgE-mediated inflammation of the nasal mucosa, resulting in eosinophilic and Th2-cell infiltration of the nasal lining • Further classified as intermittent or persistent

**Autonomic**	• Drug-induced (rhinitis medicamentosa) • Hypothyroidism • Hormonal • Non-allergic rhinitis with eosinophilia syndrome (NARES)

**Infectious**	• Precipitated by viral (most common), bacterial, or fungal infection

**Idiopathic**	• Etiology cannot be determined

**Figure 1:**
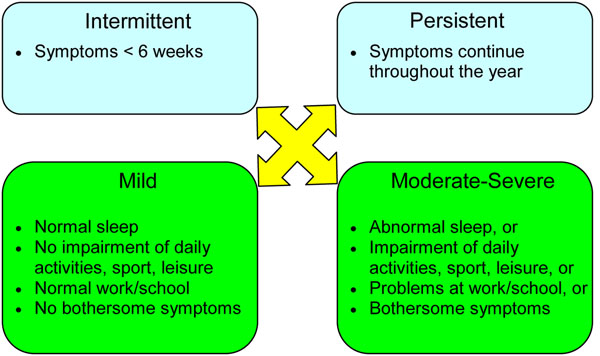
**Classification of allergic rhinitis according to symptom duration and severity.** Adapted from Small et al., 2007 [1], Bousquet et al., 2008 [5]

Traditionally, allergic rhinitis has been categorized as seasonal (occurs during a specific season) or perennial (occurs throughout the year). However, not all patients fit into this classification scheme. For example, some allergic triggers, such as pollen, may be seasonal in cooler climates, but perennial in warmer climates, and patients with multiple “seasonal” allergies may have symptoms throughout most of the year [4]. Therefore, allergic rhinitis is now classified according to symptom duration (intermittent or persistent) and severity (mild, moderate or severe) (see Figure 1) [1,5]. Rhinitis is considered intermittent when the total duration of the episode of inflammation is less than 6 weeks, and persistent when symptoms continue throughout the year. Symptoms are classified as mild when patients are generally able to sleep normally and perform normal activities (including work or school); mild symptoms are usually intermittent. Symptoms are categorized as moderate/severe if they significantly affect sleep and activities of daily living and/or if they are considered bothersome. It is important to classify the severity and duration of symptoms as this will guide the management approach for individual patients [1].

## Diagnosis and investigations

Allergic rhinitis is usually a long-standing condition that often goes undetected in the primary-care setting. Patients suffering from the disorder often fail to recognize the impact of the disorder on quality of life and functioning and, therefore, do not frequently seek medical attention. In addition, physicians fail to regularly question patients about the disorder during routine visits [1,6]. Therefore, screening for rhinitis is recommended, particularly in asthmatic patients since studies have shown that rhinitis is present in up to 95% of patients with asthma [7-10].

A thorough history and physical examination are the cornerstones of establishing the diagnosis of allergic rhinitis (see Table 2). Allergy testing is also important for confirming that underlying allergies cause the rhinitis [1]. Referral to an allergist should be considered if the diagnosis of allergic rhinitis is in question.

**Table 2:** Components of a complete history and physical examination for suspected rhinitis [**1**]

History	Physical examination
**Personal** • Nasal itch • Rhinorrhea • Sneezing • Eye involvement • Seasonality • Triggers **Family** • Allergy • Asthma **Environmental** • Pollens • Animals • Flooring/upholstery • Mould • Humidity • Tobacco exposure **Medication/drug use** • Beta-blockers • ASA • NSAIDs • ACE inhibitors • Hormone therapy • Recreational cocaine use **Quality of life** • Rhinitis-specific questionnaire **Comorbidities** • Asthma • Mouth breathing • Snoring • Sinus involvement • Otitis media • Nasal polyps • Conjunctivitis **Response to previous medications** • Second-generation oral antihistamines • Intranasal corticosteroids	**Outward signs** • Mouth breathing • Rubbing the nose/transverse nasal crease • Frequent sniffling and/or throat clearing • Allergic shiners (dark circles under eyes) **Nose** • Mucosal swelling, bleeding • Pale, thin secretions • Polyps or other structural abnormalities **Ears** • Generally normal • Pneumatic otoscopy to assess for Eustachian tube dysfunction • Valsalva’s maneuver to assess for fluid behind the ear drum **Sinuses** • Palpation of sinuses for signs of tenderness • Maxillary tooth sensitivity **Posterior oropharynx** • Postnasal drip • Lymphoid hyperplasia (“cobblestoning”) • Tonsillar hypertrophy **Chest and skin** • Atopic disease • Wheezing

ASA: acetylsalicylic acid; NSAIDs: non-steroidal anti-inflammatory drugs; ACE: angiotensin-converting enzyme; OTC: over-the-counter

Adapted from Small et al., 2007 [1]

### History

During the history, patients will often describe the following classic symptoms of allergic rhinitis: nasal congestion, nasal itch, rhinorrhea and sneezing. Allergic conjunctivitis (inflammation of the membrane covering the white part of the eye) is also frequently associated with allergic rhinitis and symptoms generally include redness, tearing and itching of the eyes [1].

An evaluation of the patient’s home and work/school environments is recommended to determine potential triggers of allergic rhinitis. The environmental history should focus on common and potentially relevant allergens including pollens, furred animals, textile flooring/upholstery, tobacco smoke, humidity levels at home, as well as other potential noxious substances that the patient may be exposed to at work or at home. The use of certain medications (e.g., beta-blockers, acetylsalicylic acid [ASA], non-steroidal anti-inflammatory drugs [NSAIDs], angiotensin-converting enzyme [ACE] inhibitors, and hormone therapy) as well as the recreational use of cocaine can lead to symptoms of rhinitis and, therefore, patients should be asked about current or recent medication and drug use [1].

The history should also include patient questioning regarding a family history of atopic disease, the impact of symptoms on quality of life and the presence of comorbidities such as asthma, mouth breathing, snoring, sleep apnea, sinus involvement, otitis media (inflammation of the middle ear), or nasal polyps. Patients may attribute persistent nasal symptoms to a “constant cold” and, therefore, it is also important to document the frequency and duration of “colds” [1].

Before seeking medical attention, patients often attempt using over-the-counter or other medications to manage their symptoms. Assessing patient response to such treatments may provide information that can aid in the diagnosis and subsequent management of allergic rhinitis. For example, symptom improvement with newer, second-generation antihistamines (e.g., desloratadine [Aerius], fexofenadine [Allegra], loratadine [Claritin]) is strongly suggestive of an allergic etiology. However, it is important to note that response to first-generation antihistamines (e.g., brompheniramine maleate [Dimetane], chlorpheniramine maleate [Chlor-Tripolon], clemastine [Tavist-1]) does not imply an allergic etiology since the anticholinergic and sedative properties of these agents reduce rhinorrhea and may improve sleep quality regardless of whether the inflammation is allergic. Previous response to intranasal corticosteroids may also be suggestive of an allergic etiology, and likely indicates that such treatment will continue to be beneficial in the future [1].

Important elements of the history for patients with suspected allergic rhinitis are summarized in Table 2.

### Physical examination

The physical examination of patients with suspected allergic rhinitis should include an assessment of outward signs, the nose, ears, sinuses, posterior oropharynx (area of the throat that is at the back of the mouth), chest and skin (see Table 2). Outward signs that may be suggestive of allergic rhinitis include: persistent mouth breathing, rubbing at the nose or an obvious transverse nasal crease, frequent sniffling or throat clearing, and allergic shiners (dark circles under the eyes that are due to nasal congestion). Examination of the nose typically reveals swelling of the nasal mucosa and pale, thin secretions. An internal endoscopic examination of the nose should also be considered to assess for structural abnormalities and nasal polyps [1].

The ears generally appear normal in patients with allergic rhinitis; however, assessment for Eustachian tube dysfunction using a pneumatic otoscope should be considered. Valsalva’s maneuver (increasing the pressure in the nasal cavity by attempting to blow out the nose while holding it shut) can also be used to assess for fluid behind the ear drum [1].

The sinus examination should include palpation of the sinuses for evidence of tenderness or tapping of the maxillary teeth with a tongue depressor for evidence of sensitivity. The posterior oropharynx should also be examined for signs of post nasal drip (mucous accumulation in the back of the nose and throat), and the chest and skin should be examined carefully for signs of concurrent asthma (e.g., wheezing) or dermatitis [1].

### Diagnostic tests

Although a thorough history and physical examination are required to establish the clinical diagnosis of rhinitis, further diagnostic testing is usually necessary to confirm that underlying allergies cause the rhinitis. Skin-prick testing is considered the primary method for identifying specific allergic triggers of rhinitis. Skin prick testing involves placing a drop of a commercial extract of a specific allergen on the skin of the forearms or back, then pricking the skin through the drop to introduce the extract into the epidermis. Within 15-20 minutes, a wheal-and-flare response (an irregular blanched wheal surrounded by an area of redness) will occur if the test is positive. Testing is typically performed using the allergens relevant to the patient’s environment (e.g., pollen, animal dander, moulds and house dust mites). A reasonable alternative to skin prick testing is the use of allergen-specific IgE tests (e.g., radioallergosorbent tests [RASTs]) that provide an *in vitro* measure of a patient’s specific IgE levels against particular allergens. However, skin prick tests are generally considered to be more sensitive and cost effective than allergen-specific IgE tests, and have the further advantage of providing physicians and patients with immediate results [1,6].

## Treatment

**Figure 2:**
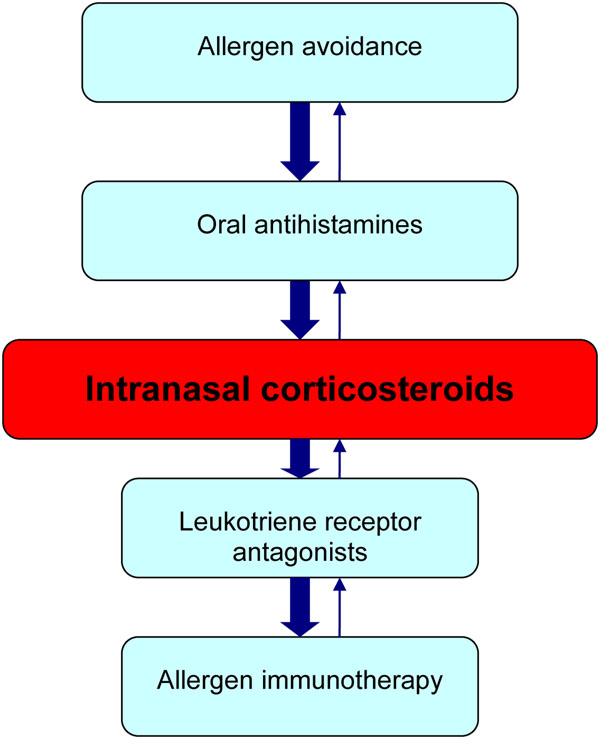
**A simplified, stepwise algorithm for the treatment of allergic rhinitis.Note: Treatments can be used individually or in any combination.**

The treatment goal for allergic rhinitis is relief of symptoms. Therapeutic options available to achieve this goal include avoidance measures, oral antihistamines, intranasal corticosteroids, leukotriene receptor antagonists, and allergen immunotherapy (see Figure 2). Other therapies that may be useful in select patients include decongestants and oral corticosteroids. If the patient’s symptoms persist despite appropriate treatment, referral to an allergist should be considered. As mentioned earlier, allergic rhinitis and asthma appear to represent a combined airway inflammatory disease and, therefore, treatment of asthma is also an important consideration in patients with allergic rhinitis.

### Allergen avoidance

The first-line treatment of allergic rhinitis involves the avoidance of relevant allergens (e.g., house dust mites, moulds, pets, pollens) and irritants (e.g., tobacco smoke). Patients allergic to house dust mites should be instructed to use allergen-impermeable covers for bedding and to keep the relative humidity in the home below 50% (to inhibit mite growth). Pollen exposure can be reduced by keeping windows closed, using an air conditioner, and limiting the amount of time spent outdoors during peak pollen seasons. For patients allergic to animal dander, removal of the animal from the home is recommended and usually results in a significant reduction in symptoms within 4-6 months. However, compliance with this recommendation is poor and, therefore, the use of high-efficiency particulate air (HEPA) filters and restricting the animal from the bedroom or to the outdoors may be needed to attempt to decrease allergen levels. Measures for reducing exposure to mould allergens include cleaning with fungicides, dehumidification to less than 50%, and HEPA filtration. These avoidance strategies can effectively improve the symptoms of allergic rhinitis, and patients should be advised to use a combination of measures for optimal results [1].

### Antihistamines

The newer, non-sedating, second-generation oral antihistamines (e.g., desloratadine [Aerius], fexofenadine [Allegra] and loratadine [Claritin]) are the first-line pharmacological treatments recommended for all patients with allergic rhinitis (see Table 3 for a list of second-generation antihistamines and their recommended dosing regimens). These agents have been found to effectively reduce sneezing, itching and rhinorrhea when taken regularly at the time of maximal symptoms or before exposure to an allergen. Although the older (first-generation) sedating antihistamines (e.g., diphenhydramine, chlorpheniramine) are also effective in relieving symptoms, they have been shown to negatively impact cognition and functioning and, therefore, they are not routinely recommended for the treatment of allergic rhinitis [1,6].

**Table 3:** Overview of pharmacologic treatment options for allergic rhinitis

	Usual adult dose	Usual pediatric dose
**Oral antihistamines (second generation)**		

Cetirizine (Reactine)	1-2 tablets (5 mg) once daily 1 tablet (10 mg) once daily	5-10 mL (1-2 teaspoons) once daily (children’s formulation)

Desloratadine (Aerius)	1 tablet (5 mg), once daily	2.5-5 mL (0.5-1.0 teaspoon) once daily (children’s formulation)

Fexofenadine (Allegra)	1 tablet (60 mg) every 12 hours (12-hour formulation) 1 tablet (120 mg), once daily (24-hour formulation)	Not currently indicated for children under 12 years of age

Loratadine (Claritin)	1 tablet (10 mg), once daily	5-10 mL (1-2 teaspoons) once daily (children’s formulation)

**Intranasal corticosteroids**		

Beclomethasone (Beconase)	1-2 sprays (42 µg/spray) EN, twice daily	1 spray (42 µg/spray) EN, twice daily

Budesonide (Rhinocort)	2 sprays (64 μg/spray) EN, once daily or 1 spray EN, twice daily	2 sprays (64 μg/spray) EN, once daily or 1 spray EN, twice daily (do not exceed 256 μg)

Ciclesonide (Omnaris)	2 sprays (50 µg/spray) EN, once daily	Not indicated for children under 12 years of age

Fluticasone furoate (Avamys)	2 sprays (27.5 µg/spray) EN, once daily	1 spray (27.5 µg/spray) EN, once daily

Fluticasone propionate (Flonase)	2 sprays (50 µg/spray) EN, once daily or every 12 hours (for severe rhinitis)	1-2 sprays (50 µg/spray) EN, once daily

Mometasone furoate (Nasonex)	2 sprays (50 µg/spray) EN, once daily	1 spray (50 µg/spray) EN, once daily

Triamcinolone acetonide (Nasacort)	2 sprays (55 µg/spray) EN, once daily	1 spray (55 µg/spray) EN, once daily

**Leukotriene receptor antagonists**		

Montelukast	1 tablet (10 mg), once daily	Not currently approved for patients under 15 years of age

EN: each nostril.

### Intranasal corticosteroids

Intranasal corticosteroids are also first-line therapeutic options for patients with mild persistent or moderate/severe symptoms and they can be used alone or in combination with oral antihistamines. When used regularly and correctly, intranasal corticosteroids effectively reduce inflammation of the nasal mucosa and improve mucosal pathology. Studies and meta-analyses have shown that intranasal corticosteroids are superior to antihistamines and leukotriene receptor antagonists in controlling the symptoms of allergic rhinitis, including nasal congestion, and rhinorrhea [11-14]. They have also been shown to improve ocular symptoms and reduce lower airway symptoms in patients with concurrent asthma and allergic rhinitis [15-17].

The intranasal corticosteroids available in Canada are shown in Table 3 and include fluticasone furoate (Avamys), beclomethasone (Beconase), fluticasone propionate (Flonase), triamcinolone acetonide (Nasacort), mometasone furoate (Nasonex), ciclesonide (Omnaris) and budesonide (Rhinocort). Since proper application of the nasal spray is required for optimal clinical response, patients should be counseled on the appropriate use of these intranasal devices. Ideally, intranasal corticosteroids are best started just prior to exposure to relevant allergens and, because their peak effect may take several days to develop, they should be used regularly [4].

The most common side effects of intranasal corticosteroids are nasal irritation and stinging. However, these side effects can usually be prevented by aiming the spray slightly away from the nasal septum [1]. Evidence suggests that intranasal beclomethasone, but not other intranasal corticosteroids, may slow growth in children compared to placebo; however, long-term studies examining the impact of intranasal beclomethasone on growth are lacking [18-21].

It is important to note that most patients with allergic rhinitis presenting to their primary-care physician have moderate-to-severe symptoms and will require an intranasal corticosteroid. Bousquet et al. noted improved outcomes in patients with moderate-to-severe symptoms treated with a combination of these agents [22].

### Leukotriene receptor antagonists

The leukotriene receptor antagonists (LTRAs) montelukast and zafirlukast are also effective in the treatment of allergic rhinitis; however, they do not appear to be as effective as intranasal corticosteroids [23-25]. Although one short-term study found the combination of LTRAs and antihistamines to be as effective as intranasal corticosteroids [26], longer-term studies have found intranasal corticosteroids to be more effective than the combination for reducing nighttime and nasal symptoms [12,27]. It is important to note that in Canada, montelukast (Singulair) is the only LTRA indicated for the treatment of allergic rhinitis in adults.

LTRAs should be considered when oral antihistamines and/or intranasal corticosteroids are not well tolerated or are ineffective in controlling the symptoms of allergic rhinitis. If combination pharmacological therapy with oral antihistamines, intranasal corticosteroids and LTRAs is not effective or is not tolerated, then allergen immunotherapy should be considered [1,6].

### Allergen immunotherapy

Allergen immunotherapy involves the subcutaneous administration of gradually increasing quantities of the patient’s relevant allergens until a dose is reached that is effective in inducing immunologic tolerance to the allergen. This form of therapy has been shown to be effective for the treatment of allergic rhinitis caused by pollens and dust mites, but has limited usefulness in treating mould and animal dander allergies [1].

Typically, allergen immunotherapy is given on a perennial basis with weekly incremental increases in dose over the course of 6-8 months, followed by maintenance injections of the maximum tolerated dose every 3 to 4 weeks for 3 to 5 years. After this period, many patients experience a prolonged, protective effect and, therefore, consideration can be given to stopping therapy. Pre-seasonal preparations that are administered on an annual basis are also available [1,6]. Sublingual preparations are also expected to be approved in Canada in the near future. These will provide patients with effective therapeutic options. Although patients will be able to self-administer the sublingual formulation, close monitoring by a physician will still be required.

Allergen immunotherapy should be reserved for patients in whom optimal avoidance measures and pharmacotherapy are insufficient to control symptoms or are not well tolerated. Since this form of therapy carries the risk of anaphylactic reactions, it should only be prescribed by physicians who are adequately trained in the treatment of allergy and who are equipped to manage possible life-threatening anaphylaxis [1].

A simplified, stepwise algorithm for the treatment of allergic rhinitis is provided in Figure 2. Note that mild, intermittent allergic rhinitis can generally be managed effectively with avoidance measures and oral antihistamines. However, as mentioned earlier, most patients presenting with allergic rhinitis have moderate-to-severe symptoms and, therefore, will require a trial of intranasal corticosteroids.

### Other therapeutic options

Oral and intranasal decongestants (e.g., pseudoephedrine, phenylephrine) are useful for relieving nasal congestion in patients with allergic rhinitis. However, the side-effect profile associated with oral decongestants (i.e., agitation, insomnia, headache, palpitations) may limit their long-term use. Furthermore, these agents are contraindicated in patients with uncontrolled hypertension and severe coronary artery disease. Prolonged use of intranasal decongestants carries the risk of rhinitis medicamentosa (rebound nasal congestion) and, therefore, these agents should not be used for more than 5 to 10 days. Oral corticosteroids have also been shown to be effective in patients with severe allergic rhinitis that is refractory to treatment with oral antihistamines and intranasal corticosteroids. [1,4].

Although not as effective as intranasal corticosteroids, sodium cromoglycate (Cromolyn) has been shown to reduce sneezing, rhinorrhea and nasal itching and is, therefore, a reasonable therapeutic option for some patients. The anti-IgE antibody omalizumab has also been shown to be effective in seasonal allergic rhinitis and asthma [1].

Surgical therapy may be helpful for select patients with rhinitis, polyposis, or chronic sinus disease that is refractory to medical treatment. Most surgical interventions can be performed under local anesthesia in an office or outpatient setting [1].

It is important to note that allergic rhinitis may worsen during pregnancy and, as a result, may necessitate pharmacologic treatment. The benefit-to-risk ratio of pharmacological agents for allergic rhinitis needs to be considered before recommending any medical therapy to pregnant women. Intranasal sodium cromoglycate can be used as a first-line therapy for allergic rhinitis in pregnancy since no teratogenic effects have been noted with the cromones in humans or animals. The first-generation antihistamines may also be considered for allergic rhinitis in pregnancy and, if required, chlorpheniramine and diphenhydramine should be recommended given their longer-term safety record. However, the patient should be warned of the risk of sedation with these medications. If an intranasal corticosteroid is required during pregnancy, beclomethasone or budesonide nasal spray should be considered as first-line therapy because of its longer safety record. Starting or increasing allergen immunotherapy during pregnancy is not recommended because of the risk of anaphylaxis to the fetus. However, maintenance doses are considered to be safe and effective during pregnancy [1].

## Conclusions

Allergic rhinitis is a common disorder that can significantly impact patient quality of life. The diagnosis is made through a comprehensive history and physical examination. Further diagnostic testing using skin-prick tests or allergen-specific IgE tests is usually required to confirm that underlying allergies cause the rhinitis. The therapeutic options available for the treatment of allergic rhinitis are effective in managing symptoms and are generally safe and well-tolerated. Second-generation oral antihistamines and intranasal corticosteroids are the mainstay of treatment for the disorder. Allergen immunotherapy as well as other medications such as decongestants and oral corticosteroids may be useful in select cases.

## Key take-home messages

• Allergic rhinitis is linked strongly with asthma and conjunctivitis.

• Allergen skin testing is the best diagnostic test to confirm allergic rhinitis.

• Intranasal corticosteroids are the mainstay of treatment for most patients that present to physicians with allergic rhinitis.

• Allergen immunotherapy is an effective immune-modulating treatment that should be recommended if pharmacologic therapy for allergic rhinitis is not effective or is not tolerated.

## Competing interests

Dr. Peter Small has received consulting fees or honoraria from GlaxoSmithKline, Graceway Pharmaceuticals, King Pharma, Merck Frosst, Novartis, and Nycomed.

Dr. Harold Kim is the past president of the Canadian Network for Respiratory Care and co-chief editor of *Allergy*, *Asthma and Clinical Immunology*. He has received consulting fees and honoraria for continuing education from AstraZeneca, GlaxoSmithKline, Graceway Pharmaceuticals, King Pharma, Merck Frosst, Novartis, and Nycomed.
